# Emotion regulation and choice of bilateral mastectomy for the treatment of unilateral breast cancer

**DOI:** 10.1002/cam4.5963

**Published:** 2023-04-21

**Authors:** Jin‐Xiao Zhang, Allison W. Kurian, Booil Jo, Bita Nouriani, Eric Neri, James J. Gross, David Spiegel

**Affiliations:** ^1^ Department of Psychology Stanford University Stanford California USA; ^2^ Departments of Medicine and of Epidemiology and Population Health Stanford University Stanford California USA; ^3^ Department of Psychiatry and Behavioral Sciences Stanford University Stanford California USA

**Keywords:** bilateral mastectomy, breast cancer, emotion regulation, fMRI, prefrontal

## Abstract

**Background:**

There has been steadily increasing use of bilateral mastectomy (BMX) in the treatment of primary breast cancer (BC). In this study, we utilized functional magnetic resonance imaging (fMRI) to examine the influence of emotion regulation on the decision of newly diagnosed BC patients to choose BMX rather than non‐BMX treatments.

**Methods:**

We recruited 123 women with unilateral BC, 61 of whom received BMX and 62 of whom received non‐BMX treatments, and 39 healthy controls. While participants were in the fMRI scanner, we showed them BC‐related and non‐BC‐negative images. In one condition, they were instructed to watch the images naturally. In another, they were instructed to regulate their negative emotion. We compared the fMRI signal during these conditions throughout the brain.

**Results:**

With non‐BC‐negative images as the baseline, BC patients showed greater self‐reported reactivity and neural reactivity to BC‐related images in brain regions associated with self‐reflection than did controls. Among the BC patients, the BMX group showed weaker activation in prefrontal emotion regulation brain regions during emotion regulation than did the non‐BMX group.

**Conclusions:**

BC patients are understandably emotionally hyper‐reactive to BC‐related stimuli and those who ultimately received BMX experience more difficulty in regulating BC‐related negative emotion than non‐BMX BC patients. These findings offer neuropsychological evidence that difficulty in managing anxiety related to the possibility of cancer recurrence is a factor in surgical treatment decision‐making and may be an intervention target with the goal of strengthening the management of cancer‐related anxiety by nonsurgical means.

**Trial Registration:**

NCT03050463.

## 1 INTRODUCTION

While the use of unilateral mastectomies in the treatment of primary breast cancer (BC) has decreased, there has been steadily increasing use of bilateral mastectomy (BMX)^1^, ^2^, ^3^, ^4^, ^5^ despite the fact that equivalent survival has been demonstrated among early‐stage BC patients treated with unilateral mastectomies or breast‐conserving treatments (non‐BMX) in randomized trials with twenty‐years of follow‐up data.^1^, ^6^, ^7^ Use of prophylactic mastectomy has been increasing at the rate of 14.3% per year and is now the choice of 33% of women under 40.^4^ BMX usually represents both treatment (for the affected breast) and prevention (for the contralateral breast), with the rare exception of BC patients with bilateral breast tumors. The causes and outcomes of the increasing trend toward BMX are unclear. Women with breast cancer do have a slightly elevated risk of a subsequent primary cancer, on the order of 1.06 [95% CI, 1.05–1.08].^8^ However, the rise of BMX may also be due to the dissemination and increased use of sensitive diagnostic tests such as magnetic resonance imaging (MRI), and genetic testing for *BRCA1* and *BRCA2* (*BRCA1/2*) and other mutations that tangibly affect cancer risk.^9^, ^10^, ^11^ Although it may be cited as a reason for choosing BMX, potentially more favorable survival after BMX appears limited to rare subgroups of BC patients like *BRCA1/2* mutation carriers and some young women with early‐stage, hormone‐receptor‐negative disease. It may be that exposure to such information has an inordinate influence on people at risk for BC, especially those with difficulty managing their emotional responses, even if they are not mutation carriers.^12^ They may overgeneralize their risk status and overestimate the preventive effect of removing an unaffected breast.

BMX is an elective procedure for a unilateral BC, because it involves more surgery than is required for treatment. Given that BMX may have detrimental effects including surgical complications and associated costs^13^, ^14^ and damage to body image and sexual activity,^5^, ^15^, ^16^ a better understanding of the factors that lead BC patients to choose this surgery is essential to optimizing the quality of cancer care. A recent study suggested that the main reason BC patients chose BMX is not because they anticipated a significant survival benefit, but rather they aimed to reduce anxiety about developing a second primary breast cancer.^17^ The survival outcome for women who choose bilateral mastectomy is no better than that for women who chose breast‐conserving treatments with radiation, across all age groups, stages, and subtypes of breast cancer.^4^ An accompanying editorial discussing these findings emphasized the role of emotions in making such decisions and the importance of providing sufficient time for them to subside before a final treatment decision is made.^18^ Anxiety is common and understandable among those recently diagnosed with BC, and in fact, anxiety disorders are the most frequent mental disorders among BC patients. Moreover, those with BC have the highest overall prevalence of mental disorders among all types of cancer.^19^

Emotional responses are complex, coordinated phenomena that lead to behavioral, cognitive, and physiological changes, activate action tendencies, and modulate feelings.^20^ During emotional reactivity, activation is seen in core limbic regions, such as the amygdala.^21^, ^22^ Emotion regulation includes the deployment of cognitive resources to alter an emotional reaction.^20^ Neuroimaging studies have found that it is also associated with activation in medial and lateral prefrontal cortex (PFC) that are implicated in executive function more broadly.^23^, ^24^ Cognition manages emotion, while emotion can impair cognition.

Thus, emotion dysregulation, involving anxiety, fear, and other negative emotions may be a major factor in decisions regarding BC faced by some 255,000 women in the United States every year. The desire to reduce understandable current anxiety by accepting major treatment side effects to avoid the future risk of regret may drive decisions that are inconsistent with actual risk reduction. We conducted a case–control study utilizing fMRI to better understand the influence of emotion regulation on the decision of newly diagnosed BC patients choosing BMX rather than unilateral mastectomies or breast‐conserving treatments (non‐BMX), with the ultimate goal of developing an intervention to enable the management of cancer‐related anxiety by nonsurgical means. The central hypotheses of the study are that: (1) BC patients choosing BMX rather than non‐BMX treatments would show more difficulty regulating the emotion elicited by unpleasant stimuli, in particular those relevant to BC, and, (2) BC patients would show greater emotional reactivity to stimuli with content relevant to BC than controls.

## 2 METHODS

### 2.1 Participants

We recruited a patient sample of 123 women diagnosed with BC within the preceding 12 months (mean 4.9 months; SD 3.5) with stage 0‐III unilateral breast cancer. We identified 33% of potential participants from Stanford Cancer Center records, 26% from the Army of Women website, 26% from social media, and the remaining 15% from other sources. Among the BC patients, 61 had undergone or later underwent BMX, and the other 62 had undergone or later received non‐BMX treatments. The recruitment of 39 healthy controls was monitored to maintain comparability in demographics. The controls were women with no history of cancer, no first‐degree relatives or 2 or more second‐degree relatives with a BC diagnosis, or any first‐ or second‐degree relatives with ovarian cancer. We identified 57% of potential control participants from a Stanford University volunteer pool, 33% from the Army of Women website, and the remaining 10% from other sources. All women (BC and control) were English‐proficient, willing to suspend intake of benzodiazepines and to undergo brain MRI, and with no contraindications to MRI imaging (e.g., ferromagnetic metal in their body). Exclusion criteria included other current or past cancers, any significant neurologic disease, current untreated psychosis or bipolar disorder, substance/alcohol abuse/dependence, current use of psychotropic medication, pregnancy, and hearing impairment. The research protocol was reviewed and approved by the Stanford Institutional Review Board (protocol number: 34959), and all subjects provided written informed consent. The details of functional and structural MRI acquisition and data preprocessing are presented in Data S1.

### 2.2 Emotion regulation task

**FIGURE 1:**
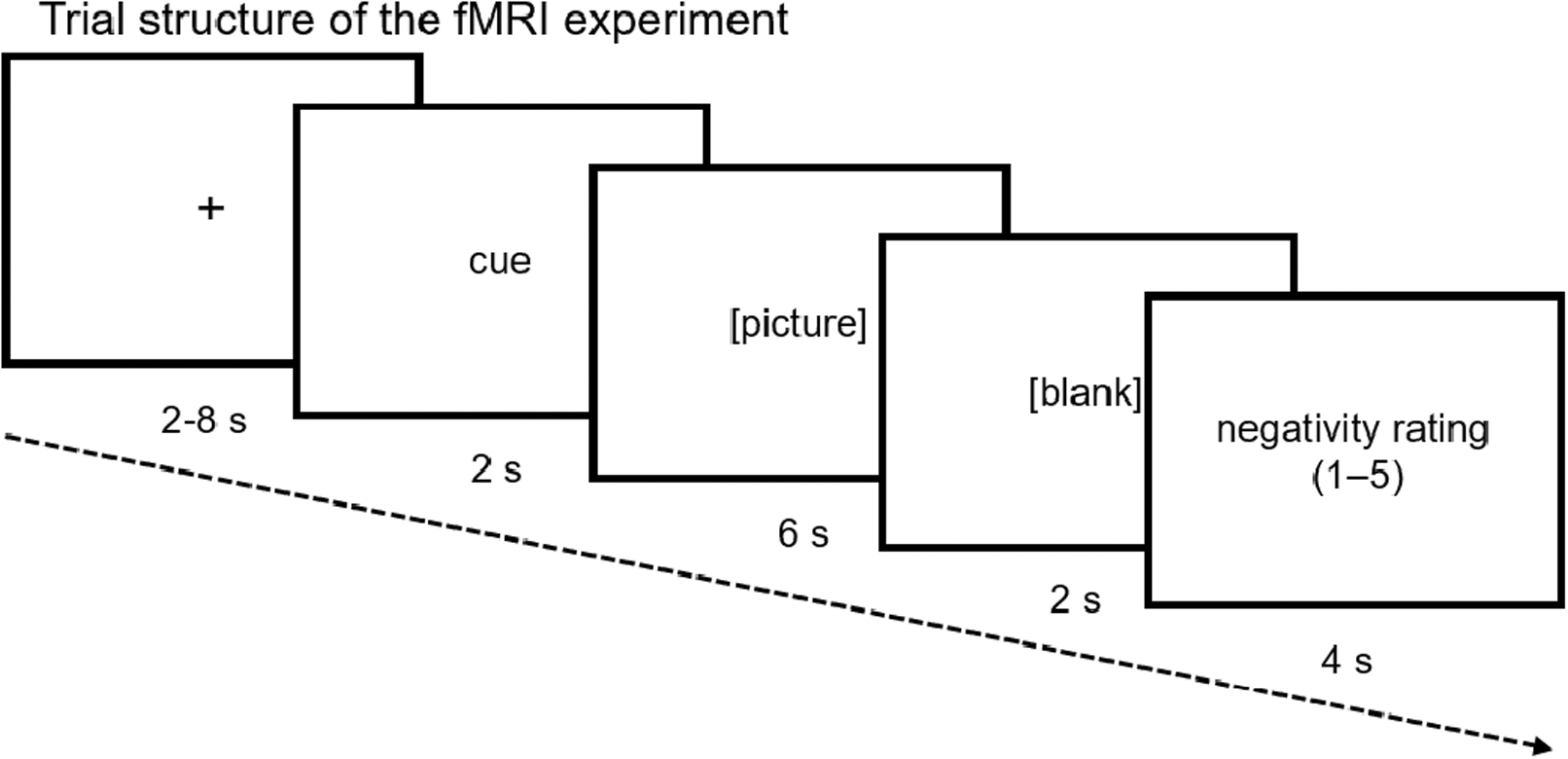
Trial structure of the emotion regulation task. A trial began with a fixation for 2–8 seconds (s), followed by a cue word “WATCH” or “RETHINK” for 2 s, a picture (neutral, IAPS‐negative, or BC‐negative) for 6 s, a blank screen for 2 s, and a rating window for 4 s.

During the task, participants viewed a series of pictures and were instructed to either respond naturally (“WATCH” condition) or to regulate their emotional response (“RETHINK” condition). Specifically, following the “WATCH” cue, they were instructed to look at and respond naturally to the picture without attempting to change their emotion. In WATCH trials, participants viewed either a neutral picture from the International Affective Picture System (IAPS),^25^ a negative picture from IAPS, or a BC‐related negative picture (e.g., breast tumor, alopecia after chemotherapy, a partially resected breast, or a body with scarring after BC surgery). In RETHINK trials, participants viewed either an IAPS or a BC‐related negative picture. Following the “RETHINK” cue (i.e., cognitive reappraisal), they were instructed to try to reinterpret the meaning of the situation depicted in the picture to feel less negative while looking at the picture (e.g., seeing BC as evidence of active and potentially effective treatment rather than as damage to one's body).^26^, ^27^ The IAPS is a widely employed set of emotionally charged pictures used to study emotion.^25^ These BC‐unrelated IAPS pictures were used as a standard comparison for the BC‐related pictures. Overall, there were 5 types of trials in the task: neutral‐watch, IAPS‐watch, BC‐watch, IAPS‐rethink, and BC‐rethink. The trial structure is illustrated in Figure 1 and more details can be found in Data S1. Particularly, at the end of a trial, participants self‐reported how negative they were feeling at the moment on a scale of 1–5 (1–not at all negative, 3–moderately negative, and 5–very much negative) using a response pad.

### 2.3 Data analysis

The study is preregistered on the Open Science Framework, and its detail can be found in the wiki pages of each of the components (https://osf.io/sq5rg/components). In particular, we preregistered the analysis plan (https://osf.io/3ju28/wiki/home/). Specifically, we preregistered the following contrasts between task conditions to index emotional reactivity: IAPS‐watch > neutral‐watch (reactivity for IAPS), BC‐watch > neutral‐watch (reactivity for BC), BC‐watch > IAPS‐watch (BC‐IAPS differential reactivity), and IAPS‐watch + BC‐watch >2 × neutral‐watch (IAPS+BC overall reactivity). Similarly, we preregistered contrasts to index emotion regulation: IAPS‐rethink > IAPS‐watch (regulation for IAPS), BC‐rethink > BC‐watch (regulation for BC), BC‐rethink > IAPS‐rethink (BC‐IAPS differential regulation), IAPS‐rethink + BC‐rethink > IAPS‐watch + BC‐watch (IAPS+BC overall regulation). We followed this logic in analyzing both the self‐report and fMRI data with the details illustrated in the subsections below. We compared these self‐report and neural indices between (1) BMX recipients versus non‐BMX recipients, and (2) controls versus BC patients. The general linear model (GLM) analyses of the fMRI data were performed using the FEAT tool in FSL 5.0.10 (https://fsl.fmrib.ox.ac.uk/fsl/fslwiki). All other statistical tests were performed in R 3.5.1 (https://www.r‐project.org/). See subsections below for the specific statistical tests performed on the fMRI data and self‐report data. Power was estimated based on comparisons between the two subgroups of breast cancer patients, with and without BMX. Given the absence of prior data on the relationship between the BMX decision and affect reactivity/regulation, we assumed a substantial but conservative effect of *d* = 0.6. Under this scenario, with the initial sample size of 120 (60 BMX, 60 non‐BMX), the estimated power to detect the association between the BMX choice and affect reactivity/regulation was 0.9. We share the fMRI and self‐report data on the OSF (https://osf.io/8tfps/).

#### 2.3.1 fMRI data

In the first‐level analysis, individual functional runs were modeled by a GLM with regressors representing the conditions including neutral‐watch, IAPS‐watch, BC‐watch, IAPS‐rethink picture, and BC‐rethink pictures (convolved with a double‐gamma function), and nuisance regressors (Data S1). The preregistered contrasts were constructed. A second‐level fixed‐effect analysis was performed to average the functional runs for each participant. The group‐level GLM analysis using a mixed‐effects method (FLAME) was performed in the whole brain to compare the contrasts between the groups. The *z* statistic images were thresholded with *z* > 3.1 voxel‐wise and a family‐wise cluster‐based correction with *p* = 0.05 in accordance with a stringent standard in the field.^28^ To control for individual difference in surgery timing, we also ran a secondary analysis that compared BMX and non‐BMX with surgery timing as a covariate (see Results for detail). Other secondary analyses included linear mixed‐effects models and Pearson's correlation that examined the relationships between fMRI and self‐report indices.

#### 2.3.2 Self‐report data

The self‐report negativity rating data of each participant were averaged across trials for each trial type. Following the preregistered analysis above (more details in Data S1), we performed independent samples t‐tests to compare the self‐report reactivity and regulation measures between (1) BMX recipients versus non‐BMX recipients, and (2) controls versus BC patients.

## 3 RESULTS

### 3.1 Sample characteristics

The sample characteristics, including demographics, are presented in Table 1. Their cancer status is presented in Table 2. Participants varied in their temporal proximity of breast cancer surgery and treatments to the fMRI experiment.

**TABLE 1:** Sample characteristics. Data are presented in a format of Mean ± SD or number (percentage).

	Controls *N* = 39 (100%)	BC patients		
		Combined *N* = 123 (100%)	BMX recipients *N* = 61 (49.6%)	Non‐BMX recipients *N* = 62 (50.4%)
Age (years)	51.1 ± 14.1	49.3 ± 10.8	47.8 ± 9.9	50.8 ± 11.6
Race (white%)	33 (84.6%)	92 (74.8%)	46 (75.4%)	46 (74.2%)
Marital status (married%)	28 (71.8%)	86 (70.0%)	51 (83.6%)	35 (56.4%)
Number of children	1.2 ± 1.1	1.6 ± 1.3	1.8 ± 1.3	1.4 ± 1.3
Education (bachelor's degree or higher%)	33 (84.6%)	93 (75.6%)	47 (77.0%)	46 (74.2%)
Income (60 K/year or higher%)	32 (82.1%)	98 (79.7%)	51 (83.6%)	47 (75.8%)
Living situation (alone%)	3 (7.7%)	13 (10.6%)	2 (3.3%)	11 (17.7%)

**TABLE 2:** Tumor status of breast cancer participants.

	Combined *N* = 123 (100%)	BMX recipients *N* = 61 (49.6%)	Non‐BMX recipients *N* = 62 (50.4%)
Cancer stage			
DCIS/LCIS	22 (17.9%)	11 (18.0%)	11 (17.7%)
Stage 1	45 (36.6%)	25 (41.0%)	20 (32.3%)
Stage 2	41 (33.3%)	15 (24.6%)	26 (41.9%)
Stage 3	15 (12.2%)	10 (16.4%)	5 (8.1%)
Tumor grade			
G1	43 (35.0%)	20 (32.8%)	23 (37.1%)
G2	36 (29.3%)	21 (34.4%)	15 (24.2%)
G3	22 (17.9%)	9 (14.8%)	13 (21.0%)
Missing	22 (17.9%)	11 (18.0%)	11 (17.7%)
Estrogen receptor status			
Positive	98 (79.7%)	50 (82.0%)	48 (77.4%)
Negative	16 (13.0%)	7 (11.5%)	9 (14.5%)
Unknown	9 (7.3%)	4 (6.6%)	5 (8.1%)
Progesterone receptor status			
Positive	86 (70.0%)	42 (68.8%)	44 (71.0%)
Negative	26 (21.1%)	14 (23.0%)	12 (19.4%)
Unknown	11 (8.9%)	5 (8.2%)	6 (9.7%)
HER2/neu status			
Positive	24 (19.5%)	13 (21.3%)	11 (17.7%)
Negative	77 (62.6%)	40 (65.6%)	37 (59.7%)
Unknown	22 (17.9%)	8 (13.1%)	14 (22.6%)
Germline *BRCA1/2* testing results			
Positive^a^	8 (6.5%)	7 (11.5%)	1 (1.6%)
Negative	94 (76.4%)	47 (77.0%)	47 (75.8%)
Not tested	20 (16.3%)	7 (11.5%)	13 (21.0%)
Missing	1 (0.8%)	0 (0.0%)	1 (1.6%)
Germline *BRCA1/2* testing in relation to treatment decision			
Before	71 (57.7%)	36 (59.0%)	35 (56.4%)
After	33 (26.8%)	18 (29.5%)	15 (24.2%)
Not tested	19 (15.5%)	7 (11.5%)	12 (19.4%)
Missing	0 (0.0%)	0 (0.0%)	0 (0.0%)

^a^ Positive for a pathogenic variant.

#### 3.1.1 Mastectomies

Forty‐six (75.4%) of participants with BMX had their BMXs before their fMRI assessments (median of 173 days, interquartile range (IQR) 119.25, 252.5); and nine (14.5%) of non‐BMX participants had their unilateral mastectomies before their fMRI assessments (median of 84 days, IQR 34, 177) (Table S2).

#### 3.1.2 Chemotherapy

Fifteen (24.6%) of participants with BMX finished chemotherapy before their fMRI assessments (median of 128 days before the fMRI, IQR 69.5, 173.5). Seven (11.3%) of non‐BMX participants finished chemotherapy before their fMRI assessments (median of 121 days before the fMRI, IQR 1126.5). Six (9.8%) of participants with BMX were receiving chemotherapy at the time of their fMRI assessments (starting median of 86 days from the fMRI, IQR 80.5, 90.75). Twelve (19.4%) of non‐BMX participants were receiving chemotherapy at the time of their fMRI assessments (starting median of 87.5 days from the fMRI, IQR 22.25, 156.25) (Table S3).

### 3.2 Emotion regulation

#### 3.2.1 fMRI data

**FIGURE 2:**
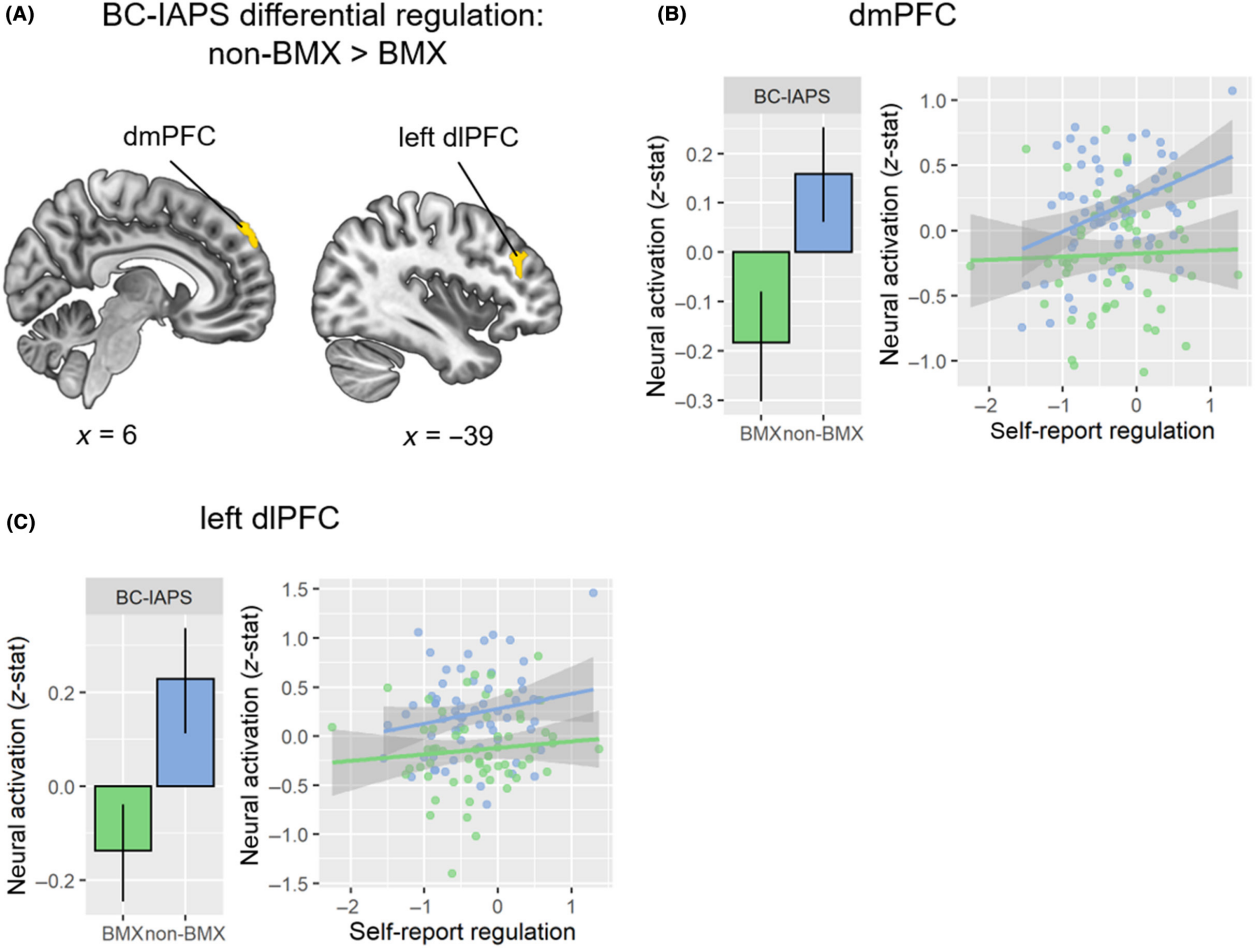
Group comparison between BMX and non‐BMX BC patients in the neural BC‐IAPS differential regulation. (A) Group comparison map of non‐BMX > BMX. The yellow regions indicate that non‐BMX recipients had greater activation for the BC‐IAPS differential regulation than BMX recipients. (B) Neural activation (*z*‐stat) for BC‐IAPS regulation in the dmPFC cluster comparing the BMX group (green) and non‐BMX group (blue). The correlations between the dmPFC neural and self‐report BC‐IAPS are consistent with our observation of differential regulation in the BMX and non‐BMX groups. The correlation in the non‐BMX group (*r* = 0.37, *p* = 0.003) was marginally stronger than that in the BMX group (*r* = 0.03, *p* = 0.784), *F*(1, 119) = 3.56, *p* = 0.062. (C) Neural activation (*z*‐stat) for BC‐IAPS regulation in the left dlPFC cluster between the BMX group (green) and non‐BMX group (blue). dlPFC, dorsolateral prefrontal cortex; dmPFC, dorsomedial prefrontal cortex. MNI coordinates in mm. Error bar: 95% confidence interval.

A BMX versus non‐BMX group comparison revealed that, for the BC‐IAPS differential regulation, the non‐BMX recipients showed greater activation in dmPFC (clusterwise *p* < 0.001) and left dlPFC (clusterwise *p* = 0.038) than BMX recipients (Figure 2A). For both dmPFC (Figure 2B) and left dlPFC (Figure 2C), the non‐BMX recipients' neural activations were significantly above 0, *p's* < 0.01, while the BMX recipients' neural activation was significantly below 0, *p's* < 0.01. Interestingly, for the dmPFC cluster, there was a marginally significant moderation effect of group (BMX vs. non‐BMX) on the relationship between the self‐report and neural indices of the BC‐IAPS differential regulation, *F*(1, 119) = 3.56, *p* = 0.062. Specifically, this self‐report‐neural correlation was medium‐sized positive in the non‐BMX group, *r* = 0.37, *p* = 0.003, while it was close to 0 in the BMX group, *r* = 0.03, *p* = 0.784 (Figure 2B). This may suggest that the dmPFC activity was linked to greater success in emotion regulation in the non‐BMX group, but that was not the case in the BMX group. This is consistent with the role of the dmPFC in managing negative emotion. For left dlPFC, the self‐report‐neural correlations did not differ between the two groups, *F*(1, 119) = 0.45, *p* = 0.505. To account for the individual difference in the time gap between BC surgery and fMRI experiment, we also ran the BMX versus non‐BMX group comparison with the surgery‐fMRI time gap as a covariate. This analysis revealed similar results, which included the same dmPFC and left dlPFC clusters (Figure S7).

There were no significant differences in the neural BC‐IAPS differential regulation between the control group and the patient group. There were no group differences between controls versus BC patients or BMX recipients versus non‐BMX recipients in other regulation contrasts (Data S1).

#### 3.2.2 Self‐report data

We compared emotion regulation between controls and BC patients and between BMX recipients and non‐BMX recipients (Figure S4A). Overall, the self‐report data did not reveal significant differences in emotion regulation between controls and BC patients or between BMX recipients and non‐BMX recipients. In addition to the preregistered analyses, we also examined trait emotion regulation reported in the Emotion Regulation Questionnaire (ERQ). Consistent with our hypothesis, we found that BMX recipients reported less use of cognitive reappraisal (i.e., the regulation strategy in the task) to regulate emotion in their daily life than non‐BMXs, *t*(111.9) = −2.38, *p* = 0.019 (Figure S4B).

### 3.3 Emotional reactivity

#### 3.3.1 fMRI data

**FIGURE 3:**
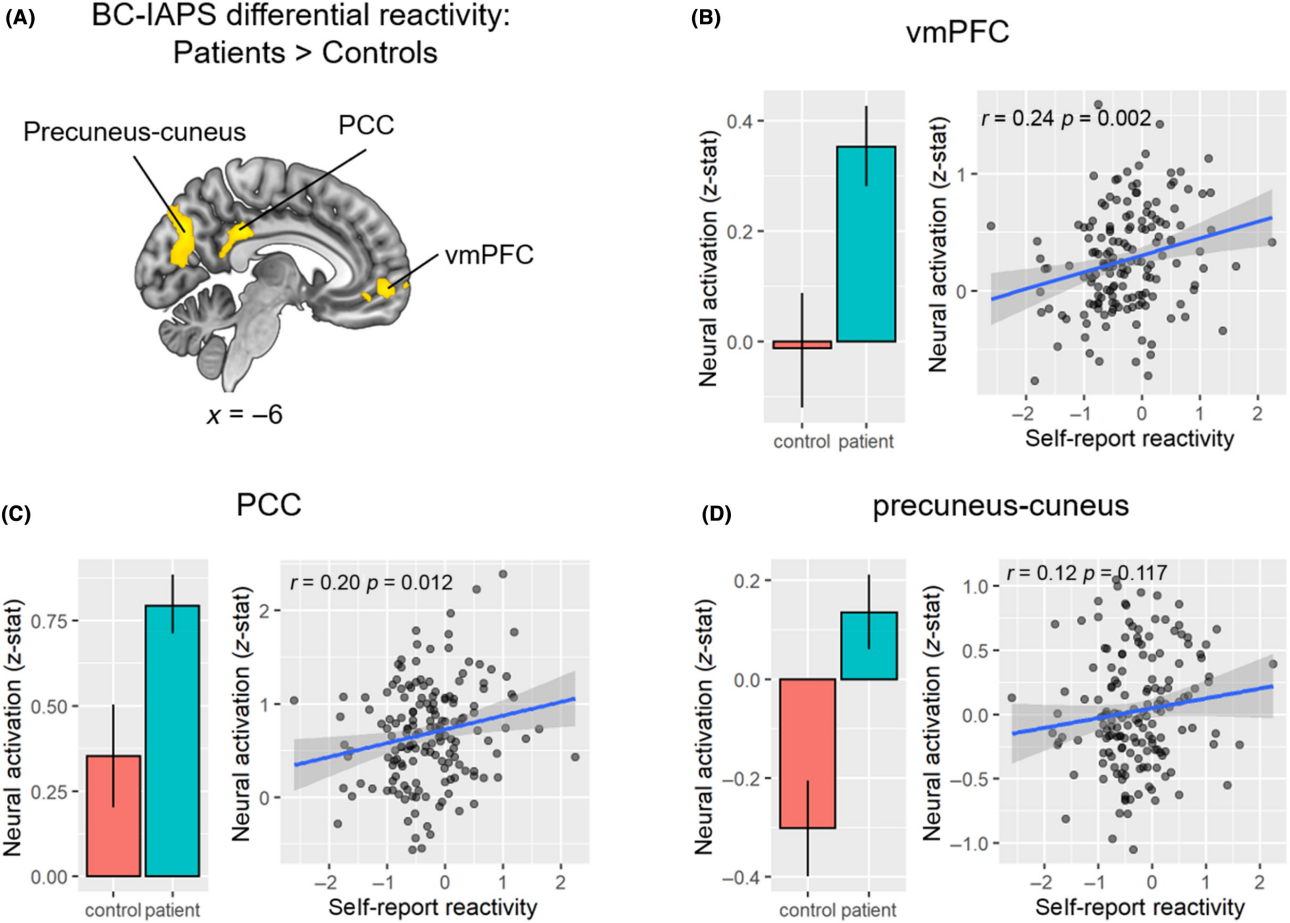
Group comparison between BC patients and controls in the neural BC‐IAPS differential reactivity. (A) Group comparison map of BC patients > controls. Yellow regions indicate where BC patients had greater activation for the BC‐IAPS differential reactivity than controls. (B) Neural activation (*z*‐stat) for BC‐IAPS reactivity in the vmPFC cluster between the control and patient groups (left) and its correlation with self‐report BC‐IAPS differential reactivity. (C) Neural activation (*z*‐stat) for BC‐IAPS reactivity in the PCC cluster between the control and patient groups (left) and its correlation with self‐report BC‐IAPS differential reactivity (right). (D) Neural activation (*z*‐stat) for BC‐IAPS reactivity in the precuneus‐cuneus cluster between the control and patient groups (left) and its correlation with self‐report BC‐IAPS differential reactivity (right). PCC, posterior cingulate cortex; Prec, precuneus; vmPFC, ventromedial prefrontal cortex. MNI coordinates in mm. Error bar: 95% confidence interval.

A patient‐control group comparison revealed that, for the BC‐IAPS differential reactivity (Figure 3A), the patient participants showed greater activation in the Default Mode Network (DMN), which processes self‐reflection,^29^ including vmPFC (clusterwise *p* = 0.005), posterior cingulate cortex (PCC, clusterwise *p* < 0.001), and a precuneus‐cuneus cluster (clusterwise *p* < 0.001) than did the healthy controls. Furthermore, the BC‐IAPS differential neural reactivity in the vmPFC and PCC clusters was significantly positively correlated with participants' self‐report of BC‐IAPS differential reactivity (vmPFC: *r* = 0.24, *p* = 0.002, Figure 3B; PCC: *r* = 0.20, *p* = 0.012, Figure 3C), supporting the idea that neural activation in these regions was related to emotional reactivity.

Within the patient group, there were no significant differences between the BMX and non‐BMX groups in the neural BC‐IAPS differential reactivity. There were no group differences between controls versus BC patients or BMX recipients versus non‐BMX recipients in other reactivity contrasts (Data S1).

#### 3.3.2 Self‐report data

As expected and consistent with the fMRI result, BC patients reported more similar emotional reactivity to BC‐related and general emotionally negative images (IAPS) than did controls, *t*(80.7) = 2.30, *p* = 0.024, while controls were more emotionally reactive to the general IAPS images than the cancer images, *t*(38) = 5.14, *p* < 0.001 (Figure S1). Among the BC patients, self‐reports of emotional reactivity did not differ significantly between the BMX group and the non‐BMX group.

## 4 DISCUSSION

The women who ultimately received BMX showed less activation in prefrontal brain regions that are associated with emotion regulation than those who received non‐BMX surgeries. Particularly, the non‐BMX group showed greater activation than the BMX group in the dmPFC and dlPFC during the application of reappraisal to regulate negative emotions elicited by BC‐related pictures.^30^, ^31^ In addition, the dmPFC activation was positively correlated with self‐reported emotion regulation success in the non‐BMX group only, suggesting that the non‐BMX group was more successful at recruiting dmPFC for emotion management than the BMX group. We further found that the non‐BMX group reported more use of cognitive reappraisal to regulate emotion in daily life than the BMX group. Overall, these findings regarding emotion regulation are consistent with our hypothesis that non‐BMX recipients appeared more successful at regulating the negative emotion elicited by BC‐related stimuli than BMX recipients.

Consistent with our hypothesis, the self‐reported BC‐IAPS differential reactivity was significantly higher in BC patients than controls. It suggests that with IAPS pictures as a baseline, BC patients reported stronger relative emotional reactivity to BC‐related pictures than their healthy counterparts. The fMRI data further corroborated this finding. When comparing the neural differential BC‐IAPS reactivity, BC patients showed significantly stronger activation in regions within the default mode network (DMN), including vmPFC, PCC, and precuneus. These brain regions are typically involved in self‐reflection and self‐evaluation processes.^32^ Moreover, neural activation in those DMN regions positively correlated with self‐report of emotional reactivity to BC and IAPS images. Our findings show that compared with healthy controls, BC patients had stronger emotional reactivity at both self‐report and neural levels toward BC‐related stimuli in particular. This confirms the salience of our measures by demonstrating neural activity underlying the anxiety related to the possibility of cancer recurrence among BC patients. These findings are consistent with studies, showing that choice of contralateral prophylactic mastectomy is primarily driven by fears of recurrence and death despite lack of evidence of improved survival.^37^

It is interesting that the BC patient‐control group difference in neural reactivity did not appear in core limbic regions such as the amygdala, but was consistently evident in several DMN regions. The amygdala typically reflects low‐level aspects of emotional reactivity. The DMN, on the contrary, is typically involved in self‐referential cognitive processes.^33^, ^34^ Our results suggest that while viewing BC‐related stimuli, these BC patients were engaged in self‐focused mental activity examining the implications of the disease for their lives. And the key difference between these BC patients and healthy controls was not their low‐level aspects of emotional reactivity, but rather their higher‐level cognitive processes that help them modulate these understandable reactions to stress. Fortunately, these cognitive processes related to fear of cancer recurrence are manageable using a variety of psychological interventions.^35^, ^36^

It is of clinical importance that greater activations of dmPFC and dlPFC during emotion regulation differentiate the non‐BMX and BMX groups. These components of the Executive Control Network (ECN) are crucial to emotion regulation, so their hypoactivation during the presentation of distressing visual stimuli, for cancer‐related images in particular, is consistent with a reduced ability to modulate negative emotion.^24^ Moreover, the correlation between self‐reported emotion regulation and activation of dmPFC was significant among the non‐BMX recipients but not among the BMX recipients. This is indicative of better ability to engage prefrontal resources to modulate the negative emotion induced by BC content. Given that the BMX procedure is more extensive, involving the removal of the noninvolved as well as the diseased breast with relatively little improvement in subsequent survival,^1^, ^6^, ^7^ these findings support the idea that difficulty in managing anxiety related to the possibility of recurrence is an important factor in the treatment decision. Our results showed that the BMX and non‐BMX groups did not differ significantly in terms of emotion reactivity but did differ in terms of emotion regulation. It suggests that, on the one hand, psychological interventions with a component of emotion regulation training (e.g., cognitive reappraisal) may be useful for BC patients. On the other hand, brain stimulation interventions (e.g., transcranial magnetic stimulation) targeting prefrontal hypoactivation in these patients could also be helpful for enhancing their ability to regulate negative emotions. It seems promising that interventions with a focus on the regulation of cancer‐related anxiety may meaningfully assist BC patients' treatment decisions by providing nonsurgical means of helping them better manage their understandable anxiety.

### 4.1 Limitations & future directions

There are several limitations to the study. First, some of our BC participants underwent the fMRI assessment after undergoing their surgical treatment. Although our secondary analysis showed that including surgery timing as a covariate did not change the main finding, future studies would benefit from assessing emotion reactivity/regulation in BC patients before they experienced their surgical treatment. Second, a small number of the BC patients were receiving chemotherapy at the time of their fMRI assessments. Although this happened in both the BMX and the non‐BMX groups, we cannot exclude the possibility that undergoing chemotherapy could have influenced participants' emotion reactivity and regulation. Third, we used BC‐related pictures to induce negative emotion but not necessarily cancer‐related anxiety in particular. Also, pictures presented in a lab setting may not fully simulate real‐life experience of cancer‐related negative emotions, although they likely stimulate affect related to the experience of women with breast cancer. Future research is encouraged to employ other paradigms to assess real‐life cancer‐related anxiety directly.

## 5 CONCLUSIONS

Employing an fMRI experiment, we found that BC patients experienced greater negative emotion in response to BC‐related stimuli than their healthy counterparts. More importantly, those who chose BMX had less activation in prefrontal regions while regulating BC‐related negative emotion than did those who chose more conservative non‐BMX treatments. These findings offer neuropsychological evidence that difficulty in managing anxiety related to cancer is a crucial factor in surgical treatment decision‐making. Difficulty in emotion regulation may serve as an intervention target with the goal of improving breast cancer treatment decision‐making by enhancing the ability to regulate cancer‐related anxiety.

## AUTHOR CONTRIBUTIONS

**Jin‐Xiao Zhang:** Formal analysis (lead); methodology (equal); visualization (lead); writing – original draft (lead); writing – review and editing (equal). **Allison Kurian:** Methodology (equal); writing – review and editing (equal). **Booil Jo:** Methodology (equal); writing – review and editing (equal). **Bita Nouriani:** Data curation (equal); methodology (equal); writing – review and editing (equal). **Eric Neri:** Data curation (equal); methodology (equal); writing – review and editing (equal). **James Gross:** Conceptualization (equal); methodology (equal); writing – review and editing (equal). **David Spiegel:** Conceptualization (equal); funding acquisition (lead); methodology (equal); writing – original draft (supporting); writing – review and editing (equal).

## CONFLICT OF INTEREST STATEMENT

The authors declare no potential conflicts of interest.

## Supporting information


Data S1.

## Data Availability

We share the neural and self‐report data on the Open Science Framework (https://osf.io/8tfps/).
