# Supplementary material for: Emotion regulation and choice of bilateral mastectomy for the treatment of unilateral breast cancer
Source: Cancer Med. 2023 Apr 21;12(11):12837–46. doi: 10.1002/cam4.5963 (PMC10278496; doi:10.1002/cam4.5963)
Supplement: Supplementary file 1 — Data S1. [file CAM4-12-12837-s001.docx]

Supplementary Material

for

**Emotion Regulation and Choice of Bilateral Mastectomy for the Treatment of Unilateral Breast Cancer**

Jinxiao Zhang, MPhil^1^*, Allison W. Kurian, MD, MSc^2^, Booil Jo, PhD^3^, Bita Nouriani, MS^3^, Eric Neri, BS^3^, James J. Gross, PhD^1^, David Spiegel, MD^3^

^1^Department of Psychology, Stanford University

^2^Departments of Medicine and of Epidemiology and Population Health, Stanford University

^3^Department of Psychiatry and Behavioral Sciences, Stanford University

*Correspondence: J. Zhang, 450 Jane Stanford Way, Building 420, Stanford University, Stanford, CA 94305; E-mail: [jinx.zhang@stanford.edu](mailto:jinx.zhang@stanford.edu)

**Table of Contents**

[Supplemental Methods 3](#_Toc102129734)

[Participants 3](#_Toc102129735)

[Procedures 5](#_Toc102129736)

[Emotion Regulation Task 6](#_Toc102129737)

[MRI Data Acquisition 6](#_Toc102129738)

[MRI Data Preprocessing 6](#_Toc102129739)

[Data Analysis 6](#_Toc102129740)

[Supplemental Results 8](#_Toc102129741)

[Emotion Reactivity 8](#_Toc102129742)

[*Self-report Data* 8](#_Toc102129743)

[*fMRI Data* 9](#_Toc102129744)

[Emotion Regulation 11](#_Toc102129745)

[*Self-report Data* 11](#_Toc102129746)

[*fMRI Data* 12](#_Toc102129747)

**Supplemental Methods**

**Participants**

A total of 163 women participated in the study. One with missing health information was excluded. Exclusion criteria were receipt of neoadjuvant or other chemotherapy because of its effects on fMRI measures of brain function; a second active cancer within the past 10 years other than squamous cell carcinoma of the skin; pregnancy; or any significant neurologic disease, including dementia, multi-infarct dementia, Parkinson's or Huntington’s disease, brain tumor, progressive supranuclear palsy, seizure disorder, subdural hematoma, multiple sclerosis, or history of significant head trauma. The final sample consisted of 162 individuals.

The controls were women with no history of cancer, no first degree relative or 2 or more second degree relatives with a BC diagnosis, or any first- or second-degree relatives with ovarian cancer. We identified 57% of potential control participants from a Stanford University volunteer pool, 33% from the Army of Women website, and the remaining 10% from other sources.  All women (BC and control) were English-proficient, not pregnant, willing to suspend intake of benzodiazepines and to undergo brain MRI, and with no contraindications to MRI imaging (e.g. ferromagnetic metal in their body). They also did not have: any significant neurologic disease, current untreated psychosis or bipolar disorder, substance/alcohol abuse/dependence, or hearing impairment.

The racial composition of the sample was 77.1% (*n* = 125) white, 11.1% (*n* = 18) Asian, 3.7% (*n* = 6) black, 1.2% (*n* = 2) Native Hawaiian or Pacific Islander, 3.1% (*n* = 5) multi-race; 3.7% (*n* = 6) of the participants did not disclose their race. All participants were informed about study procedures and gave written consent before participation. The demographic, family, and socioeconomic information for the sample is presented by groups in Table 1. There were no significant group differences in age, race, marital status, number of children, education, or income between the three groups (control, BMX, and non-BMX), all *p’s* > .05. Living situation did not differ between controls and patients, *χ*^2^(1) = 0.05, *p* = .828. Within the patients, there was a significantly larger percentage of individuals who lived alone in the non-BMX group than in the BMX group, *χ*^2^(1) = 5.36, *p* = .021.

| **Table S1. Time from BC diagnosis to fMRI in days.** | | | | | | | | | |
| --- | --- | --- | --- | --- | --- | --- | --- | --- | --- |
|  | Combined  N=123 (100%) | | | BMX Recipients  N=61 (49.6%) | | | Non-BMX Recipients  N=62 (50.4%) | | |
|  | n (%) | Median | IQR | n (%) | Median | IQR | n (%) | Median | IQR |
| Initial Diagnosis to fMRI | 123 (100%) | 181 | 87.5,  267 | 61  (100%) | 222 | 146,  296 | 62 (100%) | 139 | 66.5, 216.75 |

| **Table S2. Surgeries of BC patients.** | | | | | | | | | | | | | |
| --- | --- | --- | --- | --- | --- | --- | --- | --- | --- | --- | --- | --- | --- |
|  | Combined  N=123 (100%) | | BMX Recipients  N=61 (49.6%) | | | | | | | | Non-BMX Recipients  N=62 (50.4%) | | |
| Bilateral Mastectomy | 61 (49.6%) | | 60* (98.4%) | | | | | | | | 1** (1.6%) | | |
| Unilateral Mastectomy | 20 (16.3%) | | 3 (4.9%) | | | | | | | | 17 (27.4%) | | |
| Lumpectomy | 53 (43.1%) | | 8 (13.1%) | | | | | | | | 45 (72.6%) | | |
| Reconstruction | 63 (51.2%) | | 47 (77.0%) | | | | | | | | 16 (25.8%) | | |
| Please note some patients had more than one surgery so total procedures are higher than total n.  *Changed to Non-BMX shortly post fMRI after further discussion with medical team.  **Had BMX 15 months post fMRI due to BC diagnosis in the healthy breast. | | | | | | | | | | | | | |
| ***Time from most recent surgery to fMRI in days.*** | | | | | | | | | | | | | |
|  | Combined  N=123 (100%) | | | BMX Recipients  N=61 (49.6%) | | | | | | Non-BMX Recipients  N=62 (50.4%) | | | |
|  | n (%) | Median | IQR | n (%) | | Median | | IQR | | n (%) | Median | | IQR |
| Most Recent Surgical Procedure to fMRI | 94  (76.4%) | 99 | 53,  172 | 50  (82%) | | 107.5 | | 53.8, 172.3 | | 44  (71%) | 91.5 | | 51.75, 172.5 |
| ***Surgeries occurring before fMRI in days.*** | | | | | | | | | | | | | |
|  | Combined  N=123 (100%) | | | | BMX Recipients  N=61 (49.6%) | | | | | | Non-BMX Recipients  N=62 (50.4%) | | |
|  | n (%) | Median | IQR | | n (%) | | Median | | IQR | | n (%) | Median | IQR |
| Bilateral Mastectomy | 46  (37.4%) | 173 | 119.25, 252.5 | | 46  (75.4%) | | 173 | | 119.25, 252.5 | | 0  (0.0%) | N/A | N/A |
| Unilateral Mastectomy | 11 (8.9%) | 87 | 35,  206.5 | | 2  (3.3%) | | 282.5 | | 205.25, 359.75 | | 9  (14.5%) | 84 | 34,  177 |
| Lumpectomy | 40  (32.5%) | 111.5 | 74.5, 170.25 | | 7  (11.5%) | | 115 | | 85.5,  188.5 | | 33  (53.2%) | 108 | 64,  170 |
| Reconstruction | 48  (39.0%) | 92.5 | 44.25,  181 | | 35  (57.4%) | | 99 | | 48,  186 | | 13  (21.0%) | 87 | 36,  167 |
| ***Surgeries occurring after fMRI in days.*** | | | | | | | | | | | | | |
|  | Combined  N=123 (100%) | | | | BMX Recipients  N=61 (49.6%) | | | | | | Non-BMX Recipients  N=62 (50.4%) | | |
|  | n (%) | Median | IQR | | n (%) | | Median | | IQR | | n (%) | Median | IQR |
| Bilateral Mastectomy | 13  (10.6%) | 46 | 119,  4 | | 13  (21.3%) | | 46 | | 119,  4 | | 0  (0.0%) | N/A | N/A |
| Unilateral Mastectomy | 0  (0.0%) | N/A | N/A | | 0  (0.0%) | | N/A | | N/A | | 0  (0.0%) | N/A | N/A |
| Lumpectomy | 7  (5.7%) | 10 | 61,  8 | | 0  (0.0%) | | N/A | | N/A | | 7  (11.3%) | 10 | 61,  8 |

| **Table S3. Chemotherapy treatments of BC patients.** | | | | | | | | | |
| --- | --- | --- | --- | --- | --- | --- | --- | --- | --- |
|  | Combined | | | BMX Recipients | | | Non-BMX Recipients | | |
|  | n (%) | Median | IQR | n (%) | Median | IQR | n (%) | Median | IQR |
| No Chemo | 72  (58.5%) | N/A | N/A | 37  (60.7%) | N/A | N/A | 35  (56.5%) | N/A | N/A |
| Ended (days before fMRI) | 22  (17.9%) | 123 | 62.25,  154.5 | 15  (24.6%) | 128 | 69.5,  173.5 | 7  (11.3%) | 121 | 1,  126.5 |
| Proximity (start days before fMRI) | 18  (14.6%) | 86 | 39.5, 136.25 | 6  (9.8%) | 86 | 80.5,  90.75 | 12  (19.4%) | 87.5 | 22.25, 156.25 |
| Missing | 2  (1.6%) | N/A | N/A | 0  (0.0%) | N/A | N/A | 2  (3.2%) | N/A | N/A |
| Chemotherapy: Docetaxel +Cyclophosphamide (TC); Adriamycin + Cyclophosphamide – Taxol (AC-T); Herceptin; Taxotere; Taxol; Taxotere + Carboplatin + Herceptin + Perjeta (TCHP). | | | | | | | | | |

| **Table S4. Other treatments of BC patients.** | | | |
| --- | --- | --- | --- |
|  | Combined  N (%) | BMX Recipients  N (%) | Non-BMX Recipients  N (%) |
| Radiation | 49 (37.6%) | 11 (18.0%) | 38 (61.3%) |
| Hormonal Therapy | 65 (52.9%) | 32 (52.5%) | 33 (53.2%) |
| Hormonal therapy: Tamoxifen; Letrozole, Anastrozole; Herceptin; Exemestane; Zoladex; Triptorelin. | | | |

**Procedures**

Participants entered a magnetic resonance imaging (MRI) scanner and completed a structural scan, an 8-min resting-state scan, an emotional conflict task, and an emotion regulation task (in this order). This paper reports data from the emotion regulation task of the study and the results of other tasks will be reported in separate papers. The central hypotheses of the study are that 1) BC patients would show greater emotional reactivity to unpleasant stimuli, particularly those with content relevant to breast cancer; and 2) BC patients choosing BMX rather than breast conserving procedures (unilateral mastectomy or lumpectomy plus radiation) would show more difficulty regulating the emotion elicited by unpleasant stimuli, in particular those relevant to breast cancer. The protocol was approved by the Institutional Review Board at Stanford University.

**Emotion Regulation Task**

The emotion regulation task was programmed using E-prime software (Psychology Software Tools, Sharpsburg, PA). Participants were trained on the task by the experimenter and then completed 4 practice trials. As indicated in Figure 1C, each trial began with instruction to look at a cross at the center of the screen, followed by a cue-word indicating a “WATCH” or “RETHINK” trial. Then the picture stimulus was shown for 6 seconds. After the picture presentation, there was a blank screen for rest. At the end of a trial, participants reported how negative they were feeling at the moment on a scale of 1-5 (1–not at all negative, 3–moderately negative, 5–very much negative) using a response pad. The whole task consisted of 5 blocks of 23 pictures each (115 pictures in total). Each picture was presented only once to a given participant and their order was randomized within blocks to avoid possible context effects from a fixed order of presentation. In each block, there were 7 neutral-watch trials, 4 IAPS-watch trials, 4 BC-watch trials, 4 IAPS-rethink trials, and 4 BC-rethink trials. Each block lasted about 6.5 minutes and participants took a short break inside the scanner between blocks.

We performed manipulation checks on the emotion regulation task. As expected, the stimuli in the task elicited robust behavioral emotion reactivity among the participants (increase in negativity rating from neutral-watch to IAPS-watch trials: *Mean* = 2.38, *SD* = 0.81, *t*-test against 0: *t*(161) = 37.55, *p* < .001; increase in negativity rating from neutral-watch to BC-watch trials: *Mean* = 2.10, *SD* = 0.88, *t*-test against 0: *t*(161) = 30.32, *p* < .001). The behavioral data also indicated that participants were generally successful at regulating their negative emotion induced by the stimuli (decrease in negativity rating from IAPS-watch to IAPS-rethink trials: *Mean* = 1.10, *SD* = 0.70, *t*-test against 0: *t*(161) = 20.17, *p* < .001; decrease in negativity rating from BC-watch to BC-rethink trials: *Mean* = 0.74, *SD* = 0.63, *t*-test against 0: *t*(161) = 15.06, *p* < .001).

**MRI Data Acquisition**

The anatomical and functional imaging data were acquired using a 3T GE Discovery MR750 scanner (GE Healthcare, Chicago, IL) at the Stanford Lucas Center. A high-resolution 3D T1-weighted image was acquired for each subject (124 horizontal sections, 1.17 x 1.17 x 1 mm^3^; 256 x 256 data acquisition matrix). Functional images were acquired using a T2*-weighted, spiral pulse sequence, recording 26 sections along the z-axis at a resolution of 3.44 x 3.44 x 4.9 mm^3^ (TR = 2s, Field of View = 220 x 220 mm^2^, 64 x 64 data acquisition matrix). Each fMRI run corresponded to one experiment block. For each experimental run, 205 whole-brain volumes were recorded.

**MRI Data Preprocessing**

The anatomical and functional imaging data were preprocessed in fMRIPrep 20.0.0 (<https://fmriprep.org/en/20.0.0/>). In the preprocessing pipeline, the anatomical images were brain extracted, brain tissue segmented, and registered to MNI standard space (MNI152NLin2009cAsym) with FreeSurfer surface reconstruction. The functional images were brain extracted, head-motion corrected, reconstructed on surface, and registered to MNI standard space (MNI152NLin2009cAsym). fMRIPrep also estimated and output confounds such as the mean global signal of white matter and cerebrospinal fluid (CSF), 6 head motion parameters (translation and rotation in the x, y, and z directions), and Frame-wise Displacement (FD).

**Data Analysis**

*Self-report Data*

The self-report negativity rating data of each participant were averaged across trials for each trial type. For emotional reactivity, the increase in negativity rating from neutral-watch to IAPS-watch trials represented the self-report reactivity to IAPS pictures. The increase from neutral-watch to BC-watch trials represented the self-report reactivity for BC pictures*.* The reactivity change from IAPS to BC pictures represented the BC-IAPS differential reactivity. For emotion regulation, the decrease from IAPS-watch to IAPS-rethink trials represented the self-report regulation effect for IAPS pictures. The decrease from BC-watch to BC-rethink trials represented the self-report regulation effect for BC pictures. Similarly, the difference between them represented the BC-IAPS differential regulation.

*fMRI Data*

The fMRI data analyses were performed using the FEAT tool in FSL (<https://fsl.fmrib.ox.ac.uk/fsl/fslwiki>). In the first-level analysis, individual functional runs were spatially smoothed with a Gaussian kernel of full width at half maximum of 6mm and temporally high-pass filtered with a cutoff of 100s. The data were modeled by a General Linear Model (GLM) with regressors representing the fixation, the cue word, the neutral-watch picture, the IAPS-watch picture, the BC-watch picture, the IAPS-rethink picture, the BC-rethink picture, the rest period, the rating period, and confound regressors representing the 6 head motion parameters, the overall signals of white matter and CSF, and FD. All task regressors were convolved with a double-gamma hemodynamic response function (HRF). The 8 pre-registered contrasts were constructed. A second-level fixed-effect analysis was performed to average the functional runs for each participant. The group-level analysis using a mixed-effects method (FLAME) was performed in the whole-brain to compare the contrasts between (1) controls and BC patients, and (2) non-BMX patients and BMX patients.

**Supplemental Results**

**Emotion Reactivity**

*Self-report Data*

As pre-registered, we compared emotional reactivity between controls and BC patients and between BMX recipients and non-BMX recipients (Figure S1). Independent t-tests showed that the control group and the patient group did not differ significantly in their reactivity to IAPS pictures, *t*(64.1) = 0.76, *p* = .448, or BC pictures respectively, *t*(67.8) = -0.98, *p* = .332. Similarly, for the overall reactivity to negative pictures (IAPS+BC), the control group and the patient group did not differ significantly from one another, *t*(63.9) = 0.14, *p* = .889.

Within the patient group, independent t-tests showed that the BMX group and the non-BMX group did not differ significantly in self-report of their reactivity to IAPS pictures, *t*(120.1) = 1.02, *p* = .307, BC pictures, *t*(120.7) = 1.62, *p* = .108, or to negative pictures in general (IAPS+BC), *t*(121.0) = 1.49, *p* = .139. The BC-IAPS differential reactivity did not significantly differ between the two groups, *t*(80.7) = 0.82, *p* = .412.


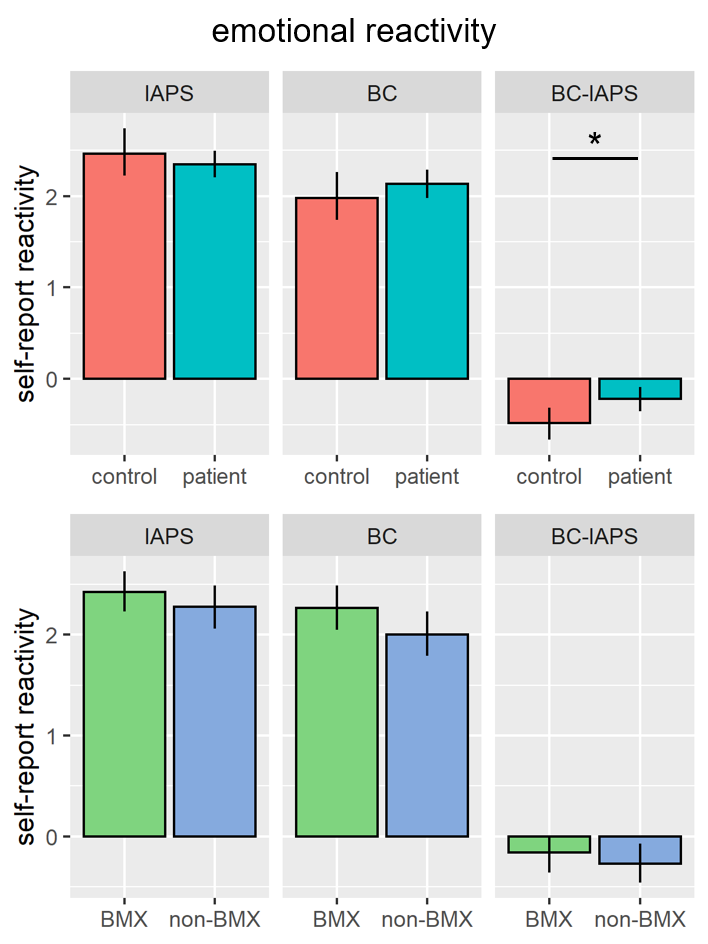


**Figure S1. Group comparisons in self-report emotional reactivity between controls vs. BC patients and BMXs vs. non-BMXs.** IAPS panels represent reactivity to IAPS pictures. BC panels represent reactivity to BC pictures. BC-IAPS represents reactivity to BC pictures with IAPS as a baseline. Patients had more similar reactivity to BC in relative to IAPS than controls, *t*(80.7) = 2.30, *p* = .024. Error bar: 95% confidence interval. **p* < .05.

*fMRI Data*

*Overall reactivity.* Across all participants, as shown in Figure S2A, the overall emotion reactivity contrast (IAPS-watch + BC-watch > 2 × neutral-watch) revealed extensive activation over the brain. We found expected activations in subcortical regions including amygdala, thalamus, putamen, and brainstem (clusterwise *p* < .001). There was also activation in parietal somatosensory association regions including superior parietal lobe, inferior parietal lobe (IPL), and supramarginal gyrus (SMG) (clusterwise *p* < .001). Activations were also found in the default mode network (DMN) regions, including the dorsomedial prefrontal cortex (dmPFC), ventromedial prefrontal cortex (vmPFC), posterior cingulate cortex (PCC), precuneus, temporal pole, and temporoparietal junction (TPJ)/angular gyrus, as well as in the Salience Network, dorsal anterior cingulate cortex (dACC) (clusterwise *p* < .001). Other activated regions included Executive Control regions: dorsolateral prefrontal cortex (dlPFC), ventrolateral prefrontal cortex (vlPFC), as well as the supplemental motor area (SMA)/pre-SMA, hand region of the somatomotor cortex (precentral and postcentral gyrus), visual cortex, and cerebellum (clusterwise *p* < .001). All the activations were similar in the left and right hemispheres. The overall emotion reactivity activation maps of control participants (Figure S2B), all patient participants (Figure S2C), or the BMX patients (Figure S2D) and non-BMX patients (Figure S2E) separately were similar to the activation map of all participants. The reactivity activation maps for IAPS (IAPS-watch > neutral-watch) and BC pictures (BC-watch > neutral-watch) respectively were largely similar to the overall reactivity map (IAPS-watch + BC-watch > 2 × neutral-watch; Figure S2A) even though a few regions showed differential activation (see BC-IAPS differential reactivity below for details). There were no significant group differences for any of those reactivity contrasts between controls vs. patients, or BMX patients vs. non-BMX patients.


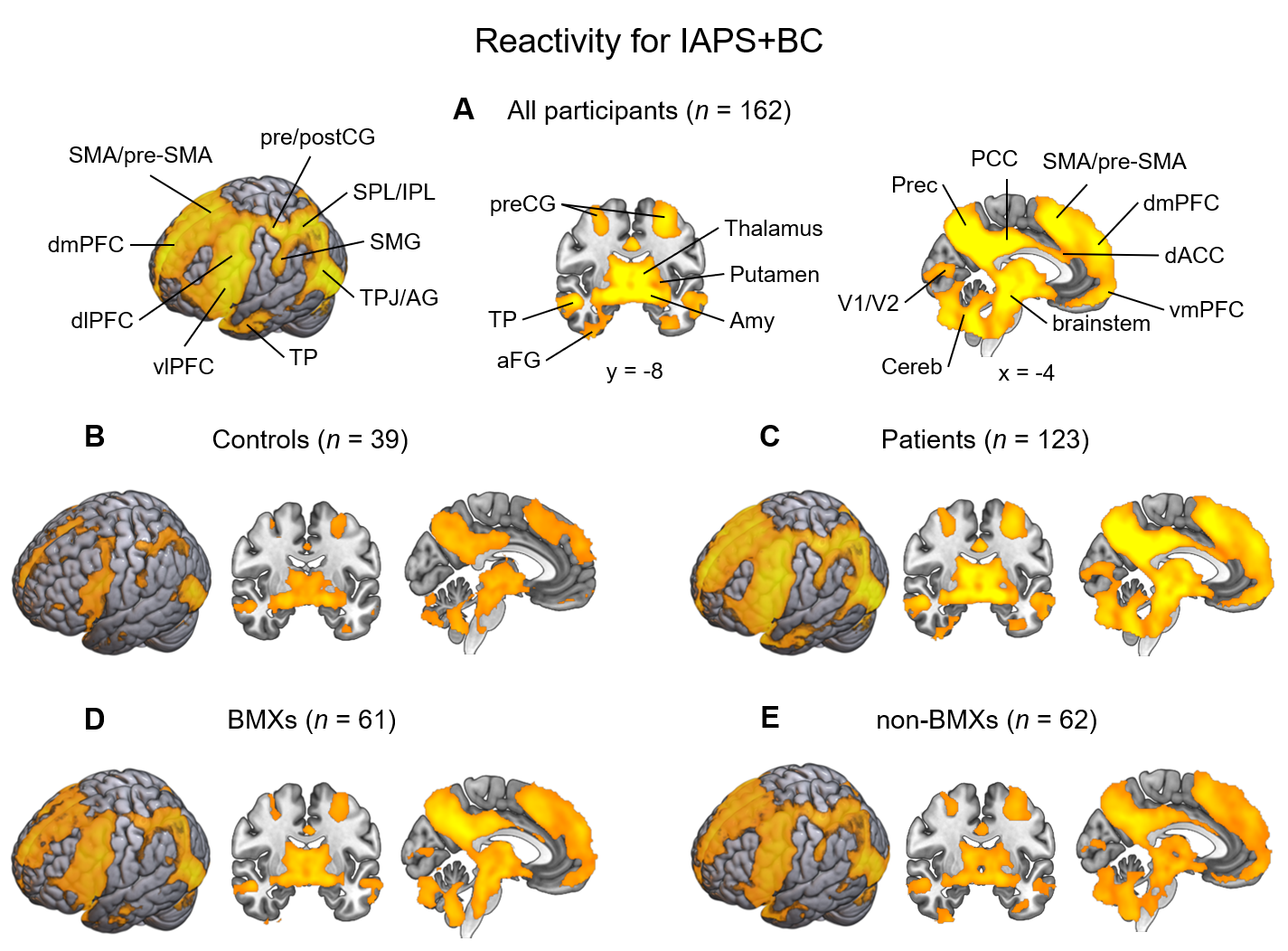


**Figure S2. Neural activation maps for overall emotion reactivity (IAPS + BC) in all participants and subgroups.** Warm colors indicate significantly stronger activation during watching negative pictures than neutral pictures. aFG, anterior fusiform gyrus; AG, angular gyrus; Amy, amygdala; Cereb, cerebellum; dACC, dorsal anterior cingulate cortex; dlPFC, dorsolateral prefrontal cortex; dmPFC, dorsomedial prefrontal cortex; IPL, inferior parietal lobe; PCC, posterior cingulate cortex; preCG, precentral gyrus; postCG, postcentral gyrus; Prec, precuneus; SMA, supplemental motor area; SMG, supramarginal gyrus; SPL, superior parietal lobe; TP, temporal pole; TPJ, temporoparietal junction; vlPFC, ventrolateral prefrontal cortex; vmPFC, ventromedial prefrontal cortex; V1, primary visual cortex; V2, secondary visual cortex. MNI coordinates in mm.

*BC-IAPS differential reactivity.* Despite the overall similarity, the BC-watch > IAPS-watch contrast revealed differences between the reactivity maps for IAPS and BC pictures (Figure S3A). Compared to IAPS pictures, BC pictures were associated with greater activations in somatosensory association regions including superior parietal lobe (SPL), inferior parietal lobe (IPL), and supramarginal gyrus (SMG), default mode network (DMN) regions including ventromedial prefrontal cortex (vmPFC), posterior cingulate cortex (PCC), precuneus, dorsal anterior cingulate cortex (dACC), and temporoparietal junction (TPJ), and a few subcortical regions including hippocampus, thalamus, and brainstem (clusterwise *p* < .05). Other regions with BC-watch > IAPS-watch activations included insula, and middle temporal gyrus (MTG) (clusterwise *p* < .05). All the activations were similar in the left and right hemispheres. We also show the BC-watch > IAPS-watch activation maps for controls (Figure S3B), all patients (Figure S3C), BMX patients (Figure S3D), and non-BMX patients (Figure S3E) separately.


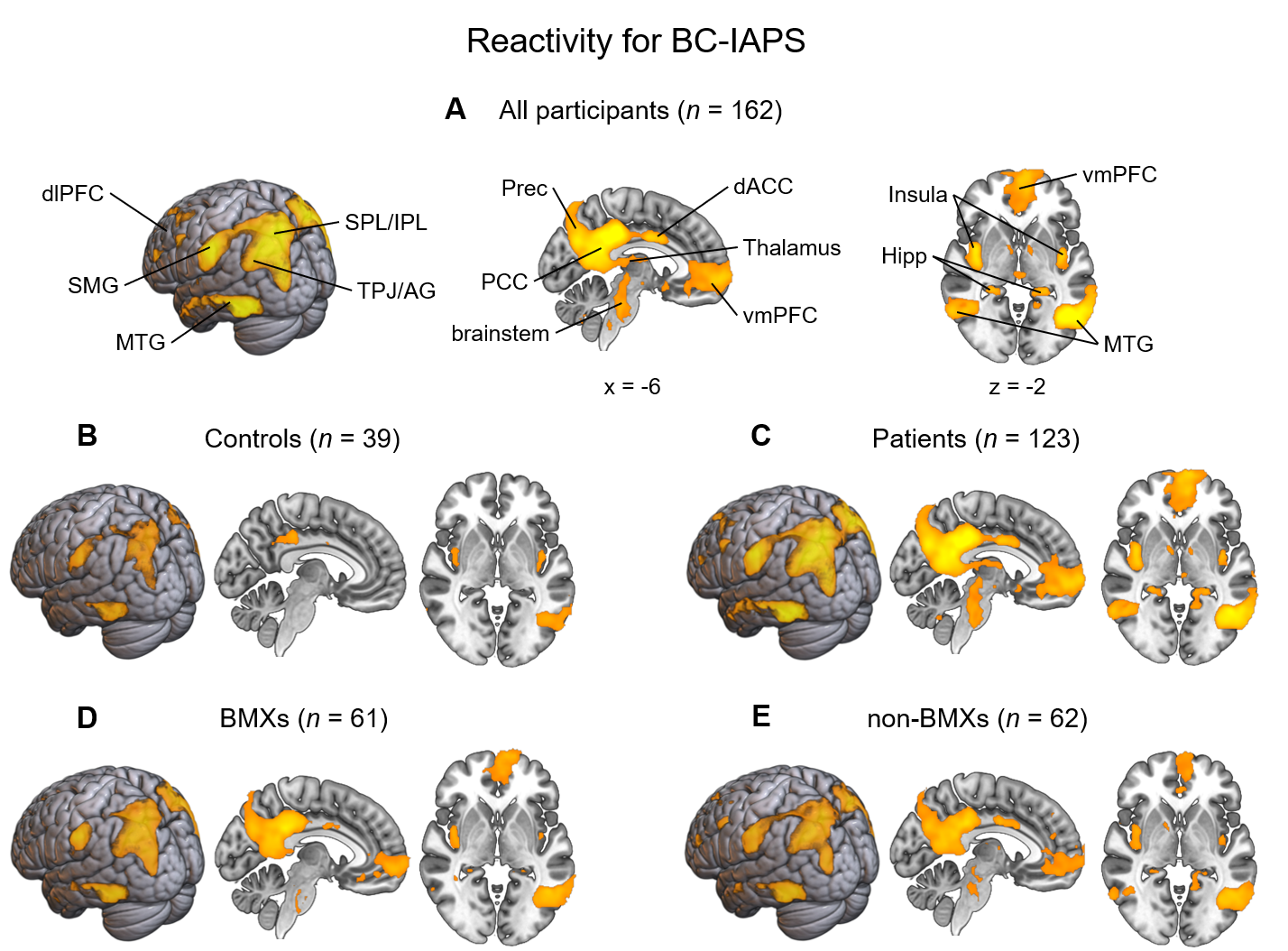


**Figure S3. Neural activation maps for differential emotion reactivity (BC - IAPS) in all participants and subgroups.** Warm colors indicate significantly stronger activation during watching breast-cancer (BC) negative pictures than IAPS negative pictures. AG, angular gyrus; dACC, dorsal anterior cingulate cortex; dlPFC, dorsolateral prefrontal cortex; IPL, inferior parietal lobe; MTG, middle temporal gyrus; PCC, posterior cingulate cortex; Prec, precuneus; SMG, supramarginal gyrus; SPL, superior parietal lobe; TPJ, temporoparietal junction; vmPFC, ventromedial prefrontal cortex. MNI coordinates in mm.

**Emotion Regulation**

*Self-report Data*

As shown in Figure S4A, there was no significant difference between the control group and the patient group in their regulation for IAPS pictures, *t*(58.8) = 1.05, *p* = .297, BC pictures, *t*(83.1) = -0.35, *p* = .726, or negative pictures overall (IAPS+BC), *t*(64.3) = 0.49, *p* = .628. The BC-IAPS differential regulation marginally differed between the control and patient groups, *t*(79.4) = -1.88, *p* = .064. However, after controlling for the BC-IAPS differential reactivity, the group effect (control vs. patient) on the BC-IAPS differential regulation became non-significant, *t*(159) = 0.25, *p* = .803.

Within the patient group, there were no significant differences between the BMX group and the non-BMX group in their regulation for IAPS pictures, *t*(120.4) = 0.31, *p* = .757, BC pictures, *t*(118.3) = 0.89, *p* = .373, or negative pictures overall (IAPS+BC), *t*(119.7) = 0.67, *p* = .503. In addition, the BMX group and the non-BMX group did not differ significantly in their BC-IAPS differential regulation, *t*(119.2) = 0.63, *p* = .528.


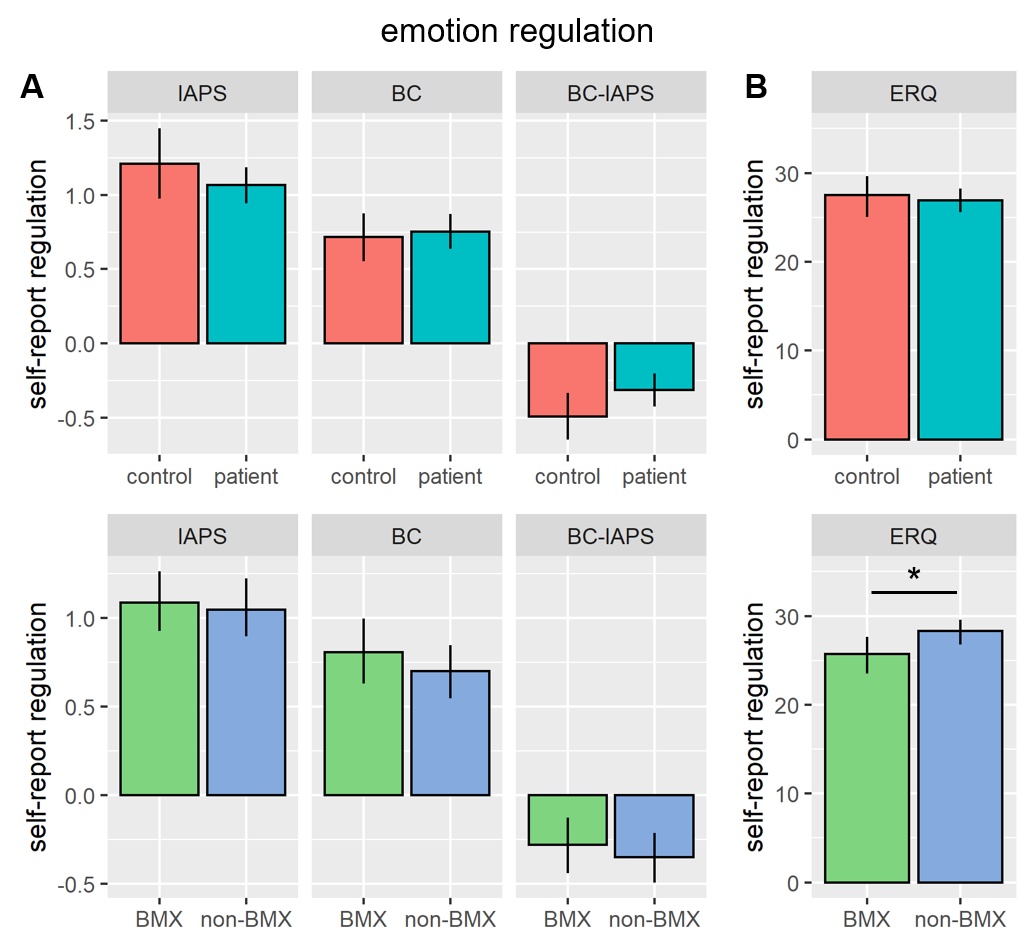


**Figure S4. Group comparisons in self-report emotion regulation between controls vs. BC patients and BMXs vs. non-BMXs.** (A) Self-report emotion regulation in task. IAPS panels represent regulation for IAPS pictures. BC panels represent regulation for BC pictures. BC-IAPS represents regulation for BC pictures minus regulation for IAPS pictures. (B) Self-report emotion regulation in Emotion Regulation Questionnaire (ERQ). The BMX group reported significantly less use of reappraisal to regulate emotion than the non-BMX group, *t*(111.9) = -2.38, *p* = .019. Error bar: 95% confidence interval. **p* < .05.

*fMRI Data*

*Overall regulation.* Across all participants, as shown in Figure S5A, the overall emotion regulation contrast (IAPS-rethink + BC-rethink > IAPS-watch+ BC-watch) revealed extensive activation over the brain. Expected activations were observed in frontoparietal regions including dmPFC, dlPFC, vlPFC, SMA/pre-SMA, dACC, TPJ/angular gyrus, and precuneus (clusterwise *p* < .001). Other activated regions included MTG, temporal pole, anterior basal ganglia regions (caudate nucleus, putamen, pallidum), visual cortex, and part of the brainstem and cerebellum (clusterwise *p* < .001). The activated regions were observed bilaterally with slightly stronger activation in the left hemisphere. The overall emotion regulation activation maps of control participants (Figure S5B), all patient participants (Figure S5C), BMX patients (Figure S5D), and non-BMX patients (Figure S5E) were similar to the activation map of all participants (Figure S5A). The regulation activation maps for IAPS (IAPS-rethink > IAPS-watch) and BC pictures (BC-rethink > BC-watch) respectively were mainly similar to the overall regulation map despite a few regions showing differential activation (see BC-IAPS differential regulation for details). As pre-registered, we performed group comparisons in those regulation contrasts between controls vs. patients, and between BMX patients vs. non-BMX patients. The only significant group difference observed was that patients’ neural activation in a small cluster surrounding the posterior right lateral sulcus (right parietal operculum and planum temporale) for the IAPS-rethink > IAPS-watch contrast was greater than the controls’ (clusterwise *p* = .046). There were no other significant group differences for those regulation contrasts.


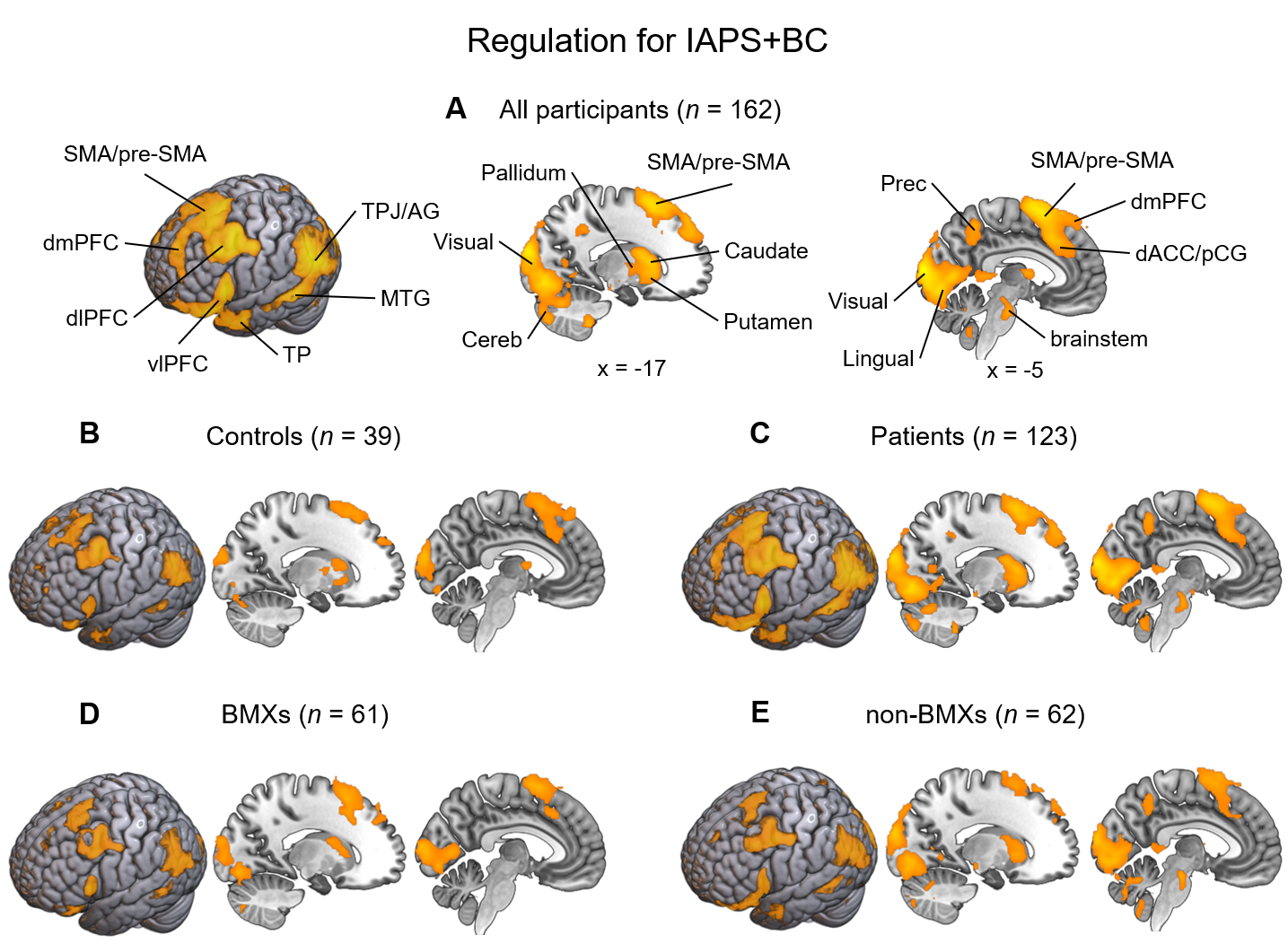


**Figure S5. Neural activation maps for overall emotion regulation (IAPS + BC) in all participants and subgroups.** Warm colors indicate significantly stronger activation during rethinking negative pictures than watching negative pictures. AG, angular gyrus; Cereb, cerebellum; dACC, dorsal anterior cingulate cortex; dlPFC, dorsolateral prefrontal cortex; dmPFC, dorsomedial prefrontal cortex; MTG, middle temporal gyrus; pCG, paracingulate gyrus; Prec, precuneus; SMA, supplemental motor area; TP, temporal pole; TPJ, temporoparietal junction; vlPFC, ventrolateral prefrontal cortex. MNI coordinates in mm.

*BC-IAPS differential regulation.* Despite the overall similarity, the BC-rethink > IAPS-rethink contrast revealed differences a few regions between the regulation maps for IAPS and BC pictures among the participants (Figure S6A). In comparison with regulation for IAPS pictures, regulation for BC pictures was associated with greater activations in frontoparietal regions including dlPFC, vlPFC, vmPFC, dACC, SPL, IPL, SMG, precuneus, and PCC (clusterwise *p* < .01). Other regions with heightened activation included insula, parahippocampal gyrus (pHG)/posterior hippocampus, MTG, inferior temporal gyrus (ITG), and lateral visual cortex (clusterwise *p* < .01). Those activated regions were observed bilaterally with slightly stronger activation in the left hemisphere. We also show the BC-rethink > IAPS-rethink activation maps for controls (Figure S6B), all patients (Figure S6C), BMX patients (Figure S6D), and non-BMX patients (Figure S6E) separately.


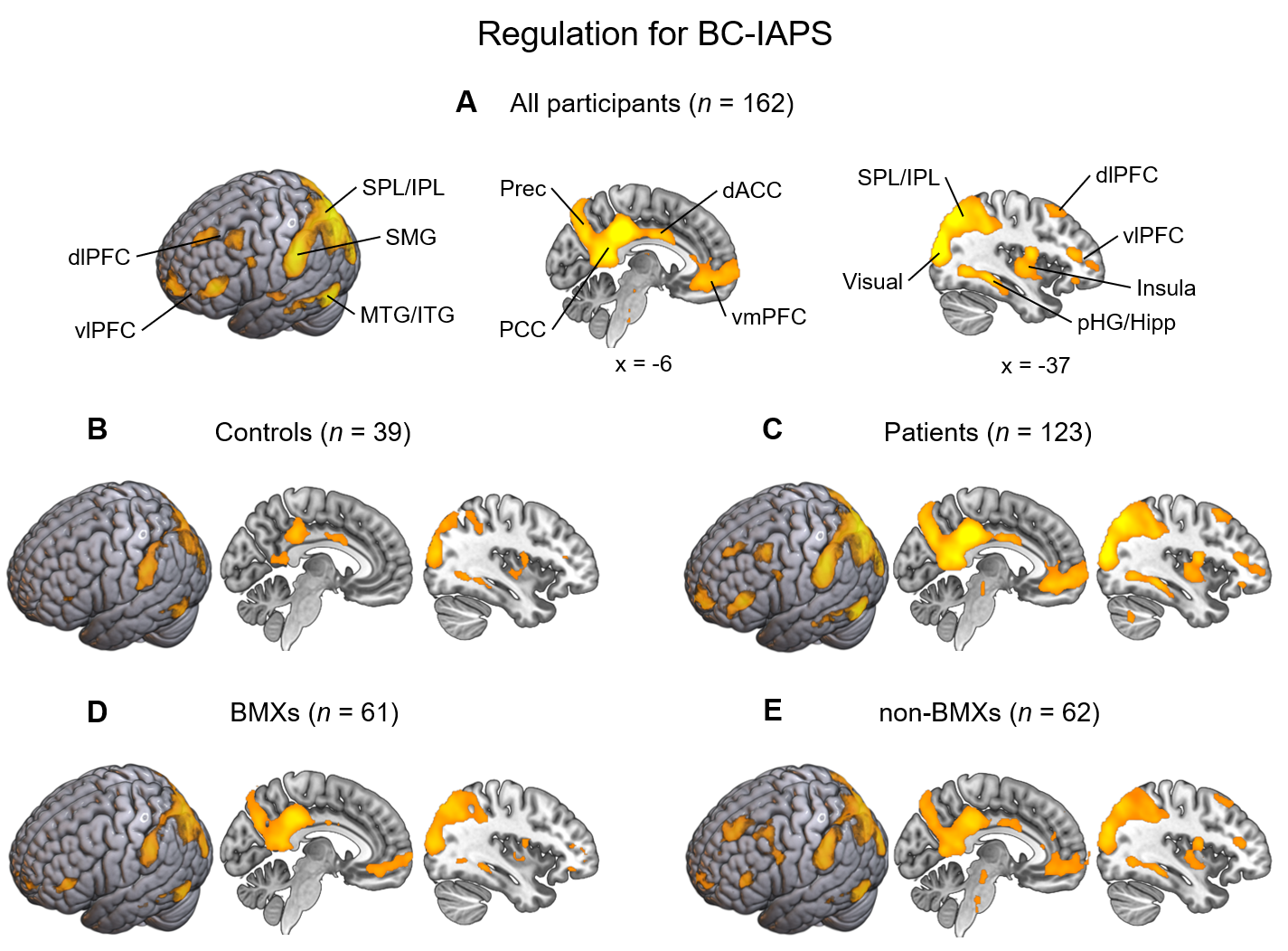


**Figure S6.** **Neural activation maps for differential emotion regulation (BC - IAPS) in all participants and subgroups.** Warm colors indicate significantly stronger activation during rethinking breast-cancer (BC) negative pictures than IAPS negative pictures. dACC, dorsal anterior cingulate cortex; dlPFC, dorsolateral prefrontal cortex; Hipp, hippocampus; IPL, inferior parietal lobe; ITG, inferior temporal gyrus; MTG, middle temporal gyrus; PCC, posterior cingulate cortex; pHG, parahippocampal gyrus; Prec, precuneus; SMG, supramarginal gyrus; SPL, superior parietal lobe; vmPFC, ventromedial prefrontal cortex; vlPFC, ventrolateral prefrontal cortex. MNI coordinates in mm.

We also compared the BC-IAPS differential regulation between the BMX and non-BMX groups while controlling for the time gap between BC surgery and fMRI experiment. The results (Figure S7) were similar to those from a t-test between BMX and non-BMX (Figure 3). In particular, it revealed that the non-BMX recipients showed greater activations than BMX recipients in dmPFC, left dlPFC, right insular cortex, and right precentral gyrus.


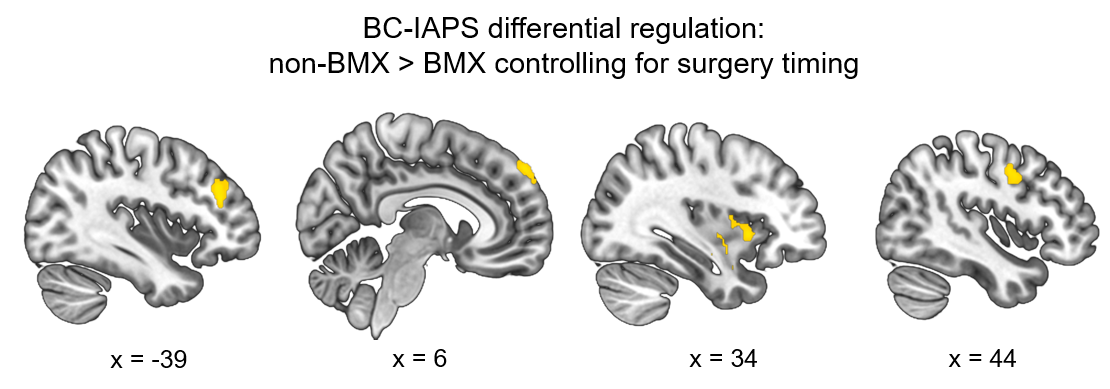


**Figure S7.** **Group comparison between BMX and non-BMX BC patients in the neural BC-IAPS differential regulation while controlling for surgery timing.** Warm colors indicate significantly stronger activation in the non-BMX group than in the BMX group.
